# Economic and Environmental Impacts of Harmful Non-Indigenous Species in Southeast Asia

**DOI:** 10.1371/journal.pone.0071255

**Published:** 2013-08-09

**Authors:** Le T. P. Nghiem, Tarek Soliman, Darren C. J. Yeo, Hugh T. W. Tan, Theodore A. Evans, John D. Mumford, Reuben P. Keller, Richard H. A. Baker, Richard T. Corlett, Luis R. Carrasco

**Affiliations:** 1 Department of Biological Sciences, National University of Singapore, Singapore, Republic of Singapore; 2 Centre for Environmental Policy, Imperial College London, London, United Kingdom; 3 Institute of Environmental Sustainability, Loyola University Chicago, Chicago, Illinois, United States of America; 4 Food and Environment Research Agency, Department for Environment, Food and Rural Affairs, York, Yorkshire, United Kingdom; 5 Xishuangbanna Tropical Botanical Garden, Chinese Academy of Sciences, Menglun, Mengla, Yunnan, China; University of Florida, United States of America

## Abstract

Harmful non-indigenous species (NIS) impose great economic and environmental impacts globally, but little is known about their impacts in Southeast Asia. Lack of knowledge of the magnitude of the problem hinders the allocation of appropriate resources for NIS prevention and management. We used benefit-cost analysis embedded in a Monte-Carlo simulation model and analysed economic and environmental impacts of NIS in the region to estimate the total burden of NIS in Southeast Asia. The total annual loss caused by NIS to agriculture, human health and the environment in Southeast Asia is estimated to be US$33.5 billion (5^th^ and 95^th^ percentile US$25.8–39.8 billion). Losses and costs to the agricultural sector are estimated to be nearly 90% of the total (US$23.4–33.9 billion), while the annual costs associated with human health and the environment are US$1.85 billion (US$1.4–2.5 billion) and US$2.1 billion (US$0.9–3.3 billion), respectively, although these estimates are based on conservative assumptions. We demonstrate that the economic and environmental impacts of NIS in low and middle-income regions can be considerable and that further measures, such as the adoption of regional risk assessment protocols to inform decisions on prevention and control of NIS in Southeast Asia, could be beneficial.

## Introduction

**Figure 1:**
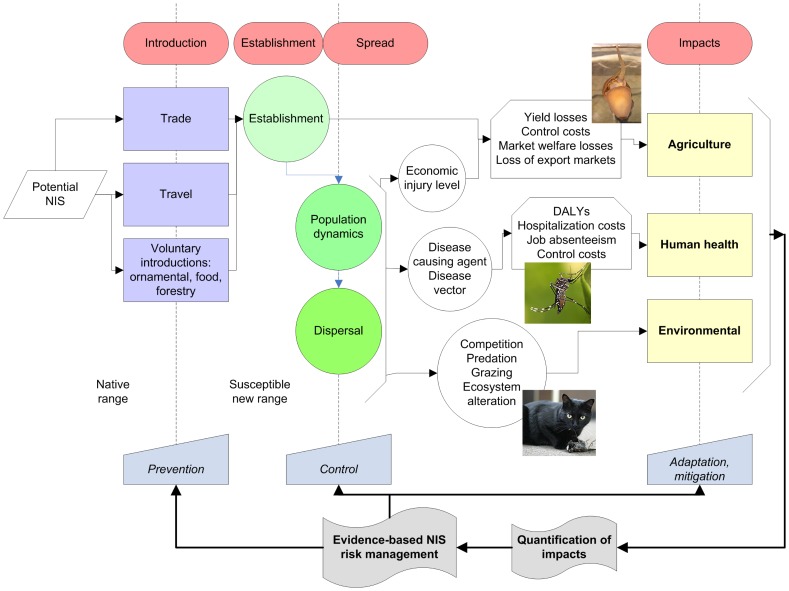
Conceptual framework of biological invasions by NIS, the impacts generated and the management measures. NIS are introduced from their native range through trade, travel or intentionally for diverse reasons such as pets or ornamental. Once established the NIS population grows and disperses. The spread of the NIS and the population levels can generate impacts to agriculture, human health and the environment. The estimation of NIS impacts is necessary to allow the generation of evidence-based risk management policies to prevent, control and mitigate the impacts of NIS in Southeast Asia. Pictures: *Pomacea caniculata* (golden apple snail), *Aedes aegypti* (dengue vector) and *Felis catus* (domestic cat). DALYs: disability-adjusted life years measures disease burden.

International trade generates wealth but it is also one of the main factors leading to the introduction of harmful non-indigenous species (NIS) [1], [2]. Especially when interacting in conjunction with habitat loss or other anthropogenic disturbances [3], NIS are one of the main threats to global biodiversity through predation, grazing, and competition with vulnerable native species [4] (Figure 1). Invasion by NIS impose enormous costs on agriculture, forestry, fisheries and water use, human health, utilities, buildings and natural areas [5]. Harmful impacts due to the introduction of NIS are likely to rise in the future as global trade increases, affecting economic welfare, environment and provisioning of ecosystem services in countries around the world [6].

Quantifying the negative impacts of NIS is complex and only a few studies have been performed that assess these impacts for multiple taxa and at regional scales. The most prominent studies have focused on the United States, where NIS are estimated to cause at least US$120 billion in economic losses per year [7]. Application of the same methods suggested that the annual environmental and economic damage from NIS equates to US$48 billion in the United Kingdom, Australia, South Africa, India, and Brazil combined [8]. In Canada, 16 NIS were estimated to cause annual economic losses of US$12–31.1 billion (2011 international dollars) [9]. Another recent study estimated that 523 NIS inflict an annual cost of US$2.5 billion (2011 international dollars) in Great Britain [10].

Although the introduction and diversity of NIS have been documented in some high-income countries, the economic and environmental impacts of NIS remain very poorly documented in middle and low-income countries in general and in Southeast Asia in particular [11]. Despite the lack of a broad knowledge about NIS in Southeast Asia, it is known that several high-impact NIS have already established and spread in the region with important ecological impacts. For example, *Mimosa pigra* is capable of outcompeting native vegetation [12], *Lantana camara* is capable of altering fire regimes [11] and the golden apple snail (*Pomacea canaliculata*) is capable of affecting ecosystem services from aquatic environments [13]. With the exception of a few NIS and countries, however, little is known about the level and types of impacts caused by NIS in Southeast Asia.

In Southeast Asia, NIS invasions are increasingly a threat to biodiversity. In Singapore, the number of known established animal NIS increased by 84% between 2003–2010 [14]. In addition, Southeast Asia contains a large share of the world’s threatened biodiversity [15] and has high rates of deforestation and forest fragmentation [16], which may render the ecosystems more susceptible and more vulnerable to invasion by NIS [17].

The scarcity of research on NIS impacts in Southeast Asia makes it impossible to fully appreciate the magnitude of the problem and hence hinders the development, adoption and coordination of evidence-based prevention and management policies. This means that opportunities to proactively identify risks and prevent the establishment of NIS – widely recognized to be the best way to reduce total damage from invasive species – are not being taken [18]. Additionally, failure to recognize the magnitude of the economic burden imposed by NIS hinders the allocation of adequate efforts and resources to manage current and future NIS invasions in the region (Figure 1). To address this critical knowledge gap, we have estimated the impacts of NIS on: (i) agricultural systems; (ii) human health; and (iii) the environment for each of the 10 member states of the Association of Southeast Asian Nations (ASEAN).

### Estimation of Impacts

All monetary values are expressed in 2011 international dollars. Purchasing power parity exchange rates [19] were used to convert local currencies into international dollars. We conducted estimation of economic impacts building on a benefit-cost analysis approach [7] and included uncertainty distributions to account for the possible uncertainties in the estimation. Monte-Carlo simulation methods were selected to propagate the uncertainty from each component to our overall estimates using @Risk [20].

### Agricultural Impacts

#### Crop pests: insects, weeds and pathogens

We estimated the economic impacts of NIS on agricultural systems in Southeast Asia by combining information on the yield losses and the proportion of NIS in major pest groups [8]. For example, in Southeast Asia, up to 46% of cassava production is lost to weeds, 22% of maize production to insects, and 22% of potato production to pathogens [21], [22] (Table S1 in File S1 contains the proportions of yield losses in major crops by pest group and proportion of NIS in each pest group). Approximately 44% and 15% of the important weeds and arthropod pests, respectively, in Southeast Asia are of non-native origin [23]. In each pest group, non-native species are not only high in number but also rated among the most damaging. Some examples includes the diamondback moth (*Plutella xylostella*) in Malaysia [24], the haganoy weed (*Chromolaena odorata*) in the Philippines [25], coffee rust (caused by the fungus *Hemileia vastatrix*) that led to the abandonment of coffee plantations in the region and ufra disease (caused by the nematode *Ditylenchus angutus*) that is one of the most important rice pathogens in Vietnam [21].

Losses from weeds, insects and pathogens were estimated as follows [7]:
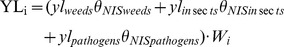
(1)where *YL_i_* represent the economic value of the yield losses in crop *i*; *yl_weeds_*, *yl_insects_*, *yl_pathogens_* are the proportions of yield losses caused by weeds, insects, and pathogens respectively; θ*_NISweeds_*, θ*_NISinsects_*_,_ θ*_NISpathogens_* are the proportions of non-indigenous weeds, insect pests, and pathogens respectively; and *W_i_* is the annual production value of crop *i* in Southeast Asia averaged over the period of 2000–2010 [26].

We applied this estimation method to a database of 101 agricultural commodities produced in Southeast Asia including food crops such as cereals (e.g. maize, rice, wheat), vegetables (e.g. pea, spinach), fruits (e.g. mango, orange, coconut) and non-food crops (e.g. rubber, cotton) [26]. The information on yield losses and the proportion of those losses that are caused by each type of NIS were only available for some crops (see File S1). For example, out of the 101 considered, yield losses by weeds were known for only 12 major crops (e.g., oil palm, rice, rubber). Therefore we could only estimate yield losses directly in 49% of the total production value of crops affected by weeds and 44% of the crops affected by insect pests. For the remaining crops, we extrapolated by constructing uncertainty distributions using the Project Evaluation and Review Techniques (PERT) distribution with the information available from the crops in each pest group (parameterized using the minimum, median (as most likely value) and maximum proportion of yield loss and proportion of NIS respectively). PERT distributions are a version of the Beta distribution that requires the same parameters as the triangular distribution. It was preferred to the triangular distribution as it does not suffer the same systematic bias problems [27]. We could not find information on the proportion of pathogens that are NIS, so we used the proportion of non-native insects as a proxy [23]. Our assumption is based on the strong association between pathogens and their insect vectors [8].

We estimated the annual total losses to crop production by non-native weeds, insects, and pathogens to be $21.6 billion (5^th^ to 95^th^ percentile: $18.06–23.05 billion). Control costs associated with weeds, insects, and pathogens were, because of data paucity, only estimated for Malaysia, Myanmar and Thailand and could not be included for the remaining countries [26]. We calculated these phytosanitary costs (*phyC_i_*) as follows:
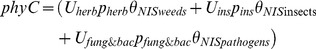
(2)where *U_herb_*, *U_ins_* and *U_fung&bac_* are respectively the usage of herbicides, insecticides, fungicides and bactericides; *p_herb_*, *p_ins_* and *p_fung&bac_* represent respectively probability distributions of the prices of these chemicals, and *ϑ*_NISweeds_, *ϑ*_NISinsects_ and *ϑ*_NISpathogens_ are the proportions of weeds, insects and pathogens that are NIS. Phytosanitary costs also include the control of pests in urban areas and golf courses.

We calculated pesticide usage by averaging annual usage data in these three countries from 2006 to 2008 [26]. Next, we estimated the cost caused by each pesticide group by constructing PERT uncertainty distributions using the annual suppliers’ price for the phytosanitary products in the Philippines as a surrogate [28]. Our estimate of the annual pesticide costs imposed by exotic weeds, insects and pathogens in the three countries for which data were available amount to $3.5 billion (5^th^ and 95^th^ percentile: $2.62–4.58 billion).

#### Molluscs: the golden apple snail

The South American mollusc, *Pomacea canaliculata,* commonly known as the golden apple snail, is a serious pest of rice fields throughout Southeast Asia. In the Philippines the annual cost of this snail to rice agriculture was estimated at $731–$2,064 million [29]. We estimated the total annual loss caused by these snails to rice in Thailand and Vietnam as $74.8 million, based on the average gross production value of rice over the last 10 years ($8.4 billion in Thailand and $10.1 billion in Vietnam; FAO 2012), proportion of surveyed locations with serious infestations of snails (density of 1 snail/m^2^ or more; 19% and 90%), and a damage ratio of 0.7% [29]. A total estimated cost of $806–$2,138 million per annum across the three countries is probably conservative because it does not include human health and environmental implications from the consumption of snails infected by disease-causing organisms (e.g., parasitic *Angiostrongylus* nematodes). It is important to note that this species has also been considered a serious rice pest in Malaysia and Indonesia where it necessitates regular interventions. However, the yield losses and control expenses in these countries have not been documented [30], [31]. In addition, the golden apple snail causes a shift in the wetland ecosystem’s state and function, thereby diminishing wetland ecosystem services across its invaded range [13].

#### Rodents

Rodents are known to raid major crops, contaminate stored grain, spread diseases, as well as compete with and prey upon native fauna [32]. Twelve important rodent species are known to cause substantial food losses to Southeast Asian countries (see File S2), two of which are exotic to Southeast Asia (*Rattus norvegicus* and *Mus musculus)*. We treated *R. rattus* as native to Southeast Asia according to recent evidence of original lineages in the *R. rattus* complex in this region [33].

We estimated the cost of rodenticides and economic damage to rice production caused by these two NIS rat species. Due to the lack of data, we calculated the amount of rodenticide used in Malaysia, Myanmar and Thailand [26] using rodenticide price from the Philippines as a surrogate [28] following a similar approach to that used for phytosanitary costs of weeds, insects and pathogens. Rice losses from 5–20% in pre-harvest and 5–10% in post-harvest are caused by rats [34]. Though the two non-native rat species are among the most destructive, we conservatively assumed that the losses are shared equally by the considered rat species in Southeast Asia. Given that total rice production of all Southeast Asian countries in the last 10 years (2001–10) averaged more than $49 billion annually, the cost of rodenticides and the loss incurred by non-native rats to rice production is estimated at $1.88 billion (5^th^ and 95^th^ percentile: $1.123–2.816 billion). Note that this estimate does not include the costs to human health caused by the diseases carried by these two NIS rodents. These diseases include: several typhus species (*Rickettsia* spp.), leptospirosis (*Leptospira*), and salmonellosis (*Salmonella*) [35].

#### Animal diseases

Foot and mouth disease is considered to be the most contagious trans-boundary disease affecting the cloven-hoofed animals that play an important role in the Southeast Asian agriculture. Annual direct losses caused by foot and mouth disease to the Philippines are estimated at $92.7 million, while the annual control costs and loss from trade restrictions in Thailand is $16.7 million [36].

Shrimp farming has expanded rapidly in Southeast Asia since the early 1990s, with an average annual production value of $4.6 billion over the last 10 years [37]. Shrimp viruses have caused substantial economic losses to farming in the region. White spot syndrome virus is considered the most serious shrimp pathogen in Asia [38] because it infects the dominant cultured shrimp species resulting in mortalities of up to 100%. Since 1994, direct losses caused by the virus to the shrimp farming industry in Asia have been as high as $1 billion per annum [39]. Since half of the shrimp production in Asia comes from Southeast Asia, we assume that half of the losses occur in Southeast Asia, i.e. $0.5 billion per year.

An outbreak of the highly pathogenic avian influenza viruses (H5N1 subtype) occurred between 2003 and 2004 in five Southeast Asian countries: Cambodia, Indonesia, Lao PDR, Thailand, and Vietnam. The outbreak resulted in the culling of 200 million poultry and a loss of over $12 billion to the poultry industry [40]. The virus has also become zoonotic and has caused 268 fatalities from 376 known infections in Southeast Asia [41]. To estimate the expected annual costs from influenza outbreaks like H5N1 we assumed a uniform distribution based on the approximately 30-year interval between influenza pandemics over the last and present century [42]. To represent the uncertainty inherent in estimating unpredictable outbreaks, we also considered half and double of this pandemic interval, and thereby estimated future influenza epidemics to range between 15 and 60 years. Our estimated annual impact based on this method was $369.7 million (5^th^ and 95^th^ percentile: $208–696 million). Our estimates are conservative taking into account the recent emergence in China of a new avian influenza H7N9 strain capable of infecting humans [43].

### Human Health Impacts

#### Measles, malaria and cholera

Although its costs in Southeast Asia could not be quantified, measles, probably of Middle Eastern origin [44], was the cause of nearly 10,500 deaths in Southeast Asia (excluding Brunei and Singapore) in 2008 alone [45]. Malaria in Southeast Asia is mostly caused by two species of *Plasmodium* (*P. falciparum* and *P. vivax*), both of which are likely to be NIS [46], [47]. Annual control costs of malaria for eight countries in Southeast Asia (excluding Brunei and Singapore) averaged for 2000–2011, to $92.8 million [48]. A new epidemic of cholera [32] could impose a heavy burden on vaccination and treatment in Southeast Asia. The cost to fully vaccinate a person is estimated at $1.10 in Vietnam [49] while the cost of illness per episode of cholera in North Jakarta is $205.7 for hospitalized cases and $28.10 for outpatient cases [50]. However, estimating the total burden of cholera in Southeast Asia was not possible because this disease is highly underreported. It is estimated that only 1% of cholera cases are reported, resulting in only 1,009 cases reported to WHO from Southeast Asia in 2012 [51].

#### Dengue illness

Dengue is a serious disease resulting in an estimated 6,000 deaths in Southeast Asia in 2008 [45]. Because there is no vaccine, the only way to prevent this disease is by controlling and reducing the breeding habitat of its primary vector, the yellow fever mosquito, *Aedes aegypti,* which originates from Africa. Dengue can have substantial economic impacts. For instance, in Singapore, the average annual cost from 2000–2009 was $88–$118 million [52] and in Thailand over a 5-year period (2001–2005) was about $390–609 million, of which 28% was allocated for vector control and 72% for illness treatment [53]. The total annual costs of dengue in the region have been estimated as $0.95 billion ($0.61–$1.38 billion) [54].

#### HIV

The human immunodeficiency virus (HIV) caused 76,750 deaths in Southeast Asia in 2008 [45]. The annual economic cost of HIV in Southeast Asia was estimated at $509.5 million, excluding Brunei for which data were not available [55]. We note that this is a conservative estimate since the individual country expenditures reported to UNAIDS are often incomplete. For example, expenditures by local governments in Thailand, the public expenditures by the non-health sectors (e.g., labour, education) in Vietnam, and expenditures on HIV treatment in public hospitals in the Philippines, were not included in this estimate.

#### SARS

The Severe Acute Respiratory Syndrome (SARS) epidemic in 2002–03 reduced Singapore’s economic growth by 1% during the course of the epidemic, resulting in a total loss of $6 billion in GDP. Malaysia suffered a $2 billion loss in tourism, food and travel sectors, while the Philippines suffered a 3% reduction in exports and trade, equivalent to a $1.5 billion loss [56]. In total, SARS cost these three Southeast Asian countries a total of $9.5 billion indirectly. Although the SARS epidemic has only emerged once, the recent outbreak of a novel human coronavirus in the Middle East (HCoV-EMC) that resembles SARS [57] suggests that other coronaviruses may have the potential to cause SARS-like epidemics in the future. We estimated the annual impact of SARS at $293 million (5^th^ and 95^th^ percentile: $164.8–551.7 million), following a similar method to that used for influenza outbreaks. This value is conservative since the direct healthcare-related costs associated with 10,000 infections with 10% mortality in the 2002–2003 epidemic in Southeast Asia were not incorporated [56].

### Environmental Damage and Other Costs

**Figure 2:**
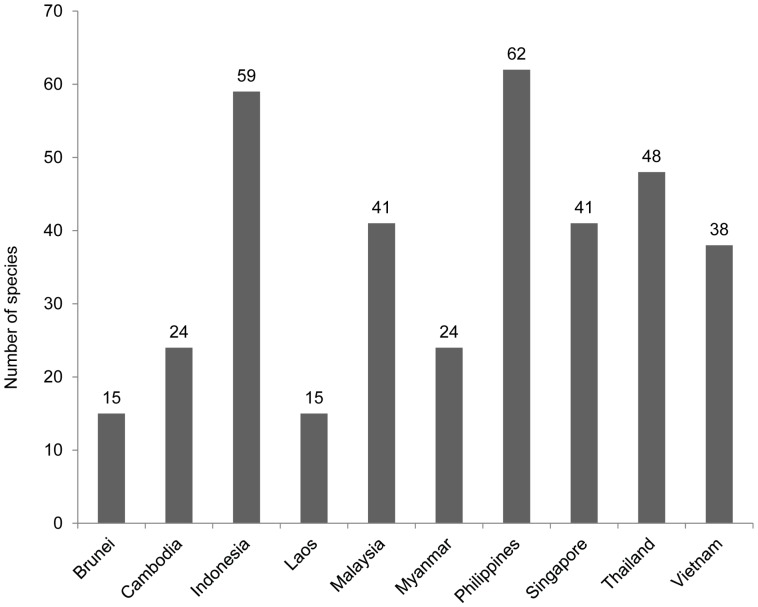
Number of reported environmental invasive species in Southeast Asian countries. Number of invasive species of environmental importance reported in 10 countries in Southeast Asia in the Global Invasive Species Database, CABI Invasive Species Compendium, Peh (2010), and MacKinnon (2006).

We compiled a list of invasive species in Southeast Asia from: (i) the Global Invasive Species Database [58]; (ii) the Invasive Species Compendium [59]; and (iii) review articles for the invasive species in the region [11], [60]. A total of 151 species was identified (Figure 2) with the highest number of species reported in the Philippines (62 species), followed by Indonesia (59 species) and the lowest number in Brunei and Laos (15 species each) (see S3 in File S1 for a comparison of the invasive species identified per country). Only four species are recorded as invasive in all 10 ASEAN countries: Siam weed (*Chromolaena odorata*), water hyacinth (*Eichhornia crassipes*), melon fruit fly (*Bactrocera cucurbitae*), and bighead carp (*Hypophthalmichthys nobilis*). Notably, 67 species are recorded as invasive in only a single ASEAN country, including the feral pigeon (*Columba livia*) recorded as invasive only in Singapore and the tree sparrow (*Passer montanus*) in the Philippines, despite the fact that these species are widely distributed across Southeast Asia [61].

Much research on NIS in Southeast Asia has been conducted with financial and technical support from organizations based outside the region (See S4 in File S1 for a list of the projects). We used data from the Australian Centre for International Agricultural Research (ACIAR), the Global Environment Facility, the Food and Agriculture Organization, and the Asian Development Bank. These organizations have collectively provided the major external funding for NIS programs in Southeast Asia. The databases of these organizations alone revealed a total budget of $11 million to support research on non-native species in Southeast Asian states annually in the last seven years. The focus of these projects varied from public health (e.g., avian influenza) and agricultural pests (e.g., Newcastle disease, leaf miner, the golden apple snail), to environmental invasive species (e.g., *C. odorata, E. crassipes*, *M. pigra*).

Environmental impacts of NIS are rarely quantified in Southeast Asia, even though they can cause irreversible damage to ecosystems. Between 1983–1999, ACIAR supported seven research projects in Indonesia, Thailand, Vietnam and the Philippines on the control of noxious invasive weeds, water hyacinth (*E. crassipes*), Siam weed (*C. odorata*), and the giant sensitive plant (*M. pigra*), with total budgets of $1.7 million, $2.3 million, and $2.9 million for each species, respectively. These three species are among the world’s worst invasive species [62] and have been recorded in most Southeast Asian countries [58], [59] where they have invaded natural, man-made (e.g., reservoirs), and agricultural systems. A series of economic impact assessments in Southeast Asia suggest that substantial environmental and agricultural losses have been caused where these species are present. Malaysia spends an estimated $1 million annually keeping irrigation systems free from water hyacinth blockages [63]. Infestations of water hyacinth in hydroelectric lakes in Thailand are also known to pose considerable costs [64]. Non-quantified losses caused by *M. pigra* include: the decline of bird species abundance by reducing diversity and abundance of wetland plant species that support bird populations in the Tram Chim National Park in Vietnam [65]; decline in fish catch, displacement of native plant species; increased agricultural land preparation expenses in Cambodia [66]; and obstruction of the irrigation system in Thailand [67].

#### Feral cats

Feral cats (*Felis catus*) are considered one of the top 100 world’s worst invaders, primarily because of their effects on biodiversity by preying on native fauna [58]. Owing to the paucity of published studies in Southeast Asia we estimated the costs caused by feral cats indirectly. Feral cat density has been found to range from 1.31–9.75 cats/km^2^, depending on food availability and conditions [68]. We assumed that feral cats would be absent from natural forests (due to competition and predation from other carnivores) and thus restricted the impacts of feral cats at a conservatively low density of 1.31 cats/km^2^ for the ca. 2.2 million km^2^ of unforested areas in Southeast Asia [26], resulting in an estimated cat population of 2.86 million. Cats are known to be extreme generalist predators, that can prey on at least 248 species including mammals, birds, amphibians, reptiles, fishes, and invertebrates, many of which are threatened [69]. Research in the US suggests that cats prey more on native than non-native bird species [70]. We conservatively narrowed the environmental impacts of cats to those resulting from killing birds which averages to 26.5 birds/cat•year [70].

We estimated the value of birds based on previous studies in which each bird can be priced from $0.37 [71] in Vietnam to $200–400 in Thailand [72]. A study of the bird trade in Singapore revealed that prayer birds can be priced at $1.40–3.60 each and songbirds at $21.40–71.30 [73]. We constructed a PERT uncertainty distribution (with the median value of the estimates as the most likely value) using these estimates. Using Monte-Carlo simulations we estimated that the cost incurred by feral cats preying on birds amounts up to $1.95 billion (5^th^ and 95^th^ percentile: 0.769–3.132 billion) annually across Southeast Asia.

#### Birds

At least 16 invasive birds have been identified in Southeast Asia. These raid grain crops, foul urban areas with faecal droppings, compete with native species, and are capable of transmitting zoonotic diseases such as avian influenza [61]. The most studied urban invaders in the region are those in Singapore. The Javan myna (*Acridotheres javanicus*) has been alleged to cause a decline in the population of native Oriental magpie robin (*Copsychus saularis*) by competing for nesting sites, in addition to its constant noise and soiling that irritates the public. The house crow (*Corvus splendens)* necessitated a $0.6 million culling campaign in 2003 in Singapore [74]. Of all the invasive birds in this region, the feral pigeon (*Columba livia*) appears to have the widest distribution. This species has colonised all 10 ASEAN countries, where it fouls structures and clogs drainages, raids crops, and is capable of transmitting 30 diseases to people, such as encephalitis and histoplasmosis [61], [74].

We estimated the damage from feral pigeons by assuming that the level of damage is similar to that in the USA, which has a density of 0.5 pigeons/person [8]. We used as a baseline the estimate from the USA of $9 per pigeon [8]. Because the costs inflicted per pigeon in Southeast Asia are not known, we used a correction factor for each Southeast Asian country calculated from the ratio of the gross national income in the USA and the country being analysed (e.g. for Thailand we assumed a maximum potential cost per pigeon of $1.54). We used these estimated values as the maximum upper bound of costs in a uniform distribution that had a lower bound of $0 to reflect our uncertainty over pigeon density in the region. We estimated an average of $146.5 million loss (5^th^ and 95^th^ percentiles: 83.8–209.3 million) incurred by feral pigeons in Southeast Asia annually. We consider our results to be conservative for urban birds since we could not quantify the economic impacts of non-native mynas in urban areas.

## Discussion

The estimated total annual losses that can be attributed to NIS in Southeast Asia is on average $33.2 billion (5^th^ and 95^th^ percentile $25.6–39.4 billion) or $55 per capita (Table 1). Because of the conservative approach adopted and the dearth of quantitative information, the actual costs are expected to be larger, especially in the case of environmental impacts through the reduction of ecosystem services that may lead to considerable economic impacts [75] and through the spill-over economic market effects. For example, a shortage in market supply of the affected commodities due to NIS invasion could lead to higher prices and consequently a reduction in consumer welfare. This could have additional impacts on industries dependent on the affected commodities. In addition, human impacts such as social instability associated with epidemics, incremental costs embedded in state management of exotic species, the loss of aesthetic value due to biological invasions and indirect environmental and health costs such as those due to pesticide usage [76], [77] could not be quantified. Although country reports suggest that NIS could pose a threat to forestry [78]–[80], we could not find sufficient data to quantify these costs in this study.

**Table 1:** Estimated annual losses caused by non-indigenous species in Southeast Asian countries ($ billion).

NIS	Mean damage (5th; 95th percentile)
***Agricultural damage***	***29.47 (23.42; 33.89)***
Weeds, insects and pathogens	21.60 (18.06; 23.05)
Pesticides	3.54 (2.62; 4.58)
Rodents (Rattus norvegicus, *Mus musculus* **)**	1.88 (1.12; 2.82)
Golden apple snail (*Pomacea canaliculata*)	1.47 (0.81; 2.14)
White spot syndrome virus	0.50
Avian influenza virus	0.37 (0.21; 0.70)
Foot and mouth disease	0.11
***Public health***	***1.85 (1.39; 2.54)***
Dengue fever	0.95 (0.61–1,38)
Human immunodeficiency virus (HIV)	0.52
Severe Acute Respiratory Syndrome (SARS)	0.29 (0.16; 0.55)
Malaria	0.09
***Environmental damage***	***2.10 (0.85; 3.34)***
Feral cat (*Felis catus*)	1.95 (0.77; 3.13)
Feral pigeon (*Columba livia*)	0.15 (0.08; 0.21)
***Project costs***	*0.01*
**Total**	**33.52 (25.78; 39.90)**

5^th^ and 95^th^ percentiles are expressed between parentheses.

In absolute value, the estimated total annual cost of $33.2 billion to Southeast Asian countries is smaller than the $120 billion damage by NIS in the USA [7]. However, in value relative to national GDP, the damage by NIS to all Southeast Asian economies (1.10–4.58%) except Singapore and Brunei is greater than in the USA, where it was 0.96% of its GDP (Table 2). Our results thus suggest that a large and poorly recognized burden of NIS may be present on low and middle-income countries.

**Table 2:** Damage costs by non-indigenous species to Southeast Asian countries ($ million).

Country	Crop pest^1^	Pesticide^2^	Rodents^3^	GAS^4^	WSSV^5^	H5N1^6^	FMD^7^	SARS	HIV	Dengue	Malaria	Feral cats^8^	Pigeons^9^	Total^10^	%GDP^11^
**Brunei**	1		<1		<1					<1		1	<1	2.6	0.02
**Cambodia**	384		62		<1	5			49	17	10	66	<4	590	4.58
**Indonesia**	7,462		599		127	261			57	323	22	769	59	9,357	1.10
**Lao PDR**	208		28			5			6	5	7	65	2	321	3.87
**Malaysia**	1,731	1,481	28		23			64	30	128	23	110	7	3,649	1.31
**Myanmar**	2,279	120	297		16				36	14	7	297	12	3,065	
**Philippines**	2,418		158	1,398	34		93	45	7	81	8	198	23	4,382	1.95
**Singapore**	0.5				<1			184	16	67		<1	1	305	0.13
**Thailand**	3,927	1,941	320	11	161	50	17		227	290	6	287	17	7,463	2.16
**Vietnam**	3,190		384	64	139	49			89	23	11	155	22	4,102	3.31

1 Calculation based on the proportional mean value to the agricultural production value which was averaged over 10 years (2001–2010) [26].

2 Calculation based on the proportion of pesticide usage in Malaysia, Myanmar and Thailand over 2006–2008 [26].

3 Rodents damage to rice production and rodenticide cost, the former was calculated as proportional to the value of rice production of each country, average over 10 years (2001–2010); the latter was calculated for three countries (Malaysia, Myanmar, Thailand) proportionate to their average usage [26]. Damage to cocoa, oil palm and coconuts could not be quantified.

4 Damage by the golden apple snail (GAS) to rice production.

5 Damage by the White Spot Syndrome Virus (WSSV) calculated as proportional to the production value of aquaculture shrimp species susceptible to the virus in each country averaged over 10 years (2001–2010) [37], susceptible species are banana shrimp (*Penaeus merguiensis)*, blue shrimp (*P. stylirostris)*, giant river prawn (*Macrobrachium rosenbergii)*, giant tiger prawn (*P. monodon)*, Kuruma prawn (*P. japonicus)*, whiteleg shrimp (*P. vannamei)* [86].

6 Damage by the avian influenza virus to the poultry industry was calculated based on the mean loss proportional to the poultry population of the countries infected in the 2003–2004 epidemic. The poultry population was averaged over 10 years (2001–2010) [26].

7 Damage by foot and mouth (FMD) disease.

8 Losses due to damage by feral cats were calculated as proportional to the land area of each country.

9 Losses due to feral pigeons were calculated as proportional to the human population of each country.

10 Project cost was not included since detailed budget allocation to beneficiary countries was unavailable.

11 Relative economic burden caused by non-indigenous species expressed as a proportion of the national GDP (except for Myanmar where GDP data are unavailable) [81].

Most of the economic impacts identified were associated with agricultural production ($29.3 billion), suggesting that countries where the agricultural sector plays an important role in economic development would be most affected. This includes all Southeast Asian states (except Singapore and Brunei) because their agricultural sectors contributed 10–50% of their GDP over the last 10 years [81]. However, economic impacts are easier to estimate for this sector because of the availability of agricultural data. There may be higher economic losses in human health and the losses of ecosystem services caused by NIS, but data on these losses are in most cases not available.

Preferably, to estimate agricultural impacts by NIS, the characterization of the markets involved for the estimation of the losses of consumers’ and producers’ surpluses would be necessary. Data paucity prevented us from characterizing the behaviour of the demand and supply curves for each of the 101 agricultural products considered in all the countries. Instead, prices times quantities were used as proxies of the economic impacts. This approach is common in large-scale studies where data paucity is present and implies assuming a vertical supply curve and a horizontal demand curve [7], [82]. As more data become available, further research could relax this assumption and attempt to estimate the surplus losses generated by NIS.

The introduction and establishment of many of the most impactful NIS in Southeast Asia are a direct consequence of intentional human activities. A typical example is the golden apple snail, which was first introduced to Southeast Asia as a source of protein. In Vietnam, this snail was initially cultured in large-scale snail farms for just a short time before it was found to be unpopular as human food. As containment measures failed, the species became a voracious pest that withstood eradication efforts [83]. The problems with the golden apple snail may have been prevented if a risk assessment had preceded its introduction and mass rearing. Despite the substantial negative consequences derived from the introduction of the golden apple snail, many other NIS that cause negative impacts elsewhere in the world are being cultivated on a large scale in Southeast Asia. For example, Australian *Eucalyptus* and *Acacia* species are grown widely in the region even though they have been identified as invasive species capable of transforming native forest ecosystems, especially on islands [60], [84]. Therefore, one main lesson from the deliberate introduction of NIS such as the golden apple snail is that preventive steps should be taken so that future expenses can be minimized.

The presence of a large number of NIS causing environmental impacts in Southeast Asia (Figure 2) suggests that the value lost through impacts on ecosystems might be comparable to that estimated for agricultural systems. A total of 151 invasive species has been reported as invasive in at least one Southeast Asian country from four regional reviews and databases [11], [58]–[60]. However, 48% of these species are only listed as invasive in one of the references (see a comparison of the list of NIS for each country from the different sources in File S1). This limitation might be caused by the heterogeneous nature of Southeast Asia, as a region with countries of different development levels, management schemes, and languages, which could hinder effective information-sharing among countries. As a result, the documentation of NIS impacts across Southeast Asian countries is less consistent than previous research focusing on single high-income countries. Given the close proximity of and connectivity among Southeast Asian countries, it is important that countries cooperate with each other to prevent the introduction and spread of NIS.

Through the recognition of substantial economic and environmental burdens that NIS impose in Southeast Asia, and in order to minimize the economic impacts of NIS, an agreed risk assessment protocol and tighter regional screening procedures should be developed and applied. Regional risk assessment protocols could provide the basis for management with positive net economic returns [85], especially in the light of the high economic impacts that NIS cause to Southeast Asia.

## Supporting Information

File S1 **Supplementary online material.**
(DOCX)
