# Supplementary material for: Economic and Environmental Impacts of Harmful Non-Indigenous Species in Southeast Asia
Source: PLoS One. 2013 Aug 9;8(8):e71255. doi: 10.1371/journal.pone.0071255 (PMC3739798; doi:10.1371/journal.pone.0071255)
Supplement: File S1 — Supplementary online material. (DOCX) [file pone.0071255.s001.docx]

**Supplementary Online Material**

**Economic and environmental impacts of harmful non-indigenous species in Southeast Asia**

Le T.P. Nghiem^1^, Tarek Soliman^1^, Darren C.J. Yeo^1^, Hugh T.W. Tan^1^, Theodore A. Evans^1^, John D. Mumford^2^, Reuben P. Keller^3^, Richard H.A. Baker^4^, Richard T. Corlett^5^, Luis R. Carrasco^1,*^

^1^ Department of Biological Sciences, National University of Singapore, Singapore, Republic of Singapore; ^2^ Centre for Environmental Policy, Imperial College London, London, UK; ^3^ Institute of Environmental Sustainability, Loyola University Chicago, Chicago, Illinois, United States of America; ^4^ Food and Environment Research Agency, Department for Environment, Food & Rural Affairs, York, Yorkshire, UK; ^5^ Xishuangbanna Tropical Botanical Garden, Chinese Academy of Sciences, Menglun, Mengla, Yunnan, China.^*^Email: dbsctlr@nus.edu.sg. Tel: +6591377291. Fax: +65 67792486.

**Table S1.** Data input for the estimation of crop loss due to NIS pests

| Weeds | | | | Insect pests | | | | Pathogens | |
| --- | --- | --- | --- | --- | --- | --- | --- | --- | --- |
| Crop | Yield loss (%)$^$ | Crop | % exotic* | Crop | Yield loss (%)^ | Crop | % exotic* | Crop | Yield loss (%)^ |
| Barley | 17 | Cotton | 36 | Cotton | 19 | Soybean | 46 | Cotton | 10 |
| Cotton | 13 | Soybean | 50 | Soybean | 13 | Potato | 50 | Soybean | 21 |
| Soybean | 13 | Cocoa | 64 | Potato | 18 | Coffee | 27 | Potato | 22 |
| Potato | 12 | Coffee | 55 | Coffee | 13 | Sugarcane | 16 | Coffee | 11 |
| Coffee | 11 | Vegetables | 75 | Maize | 22 | Cassava | 31 | Maize | 16 |
| Cassava | 46^#^ | Sugarcane | 60 | Rice^+^ | 18.5 | Maize | 24 | Rice | 15 |
| Maize | 20 | Cassava | 92 |  |  | Banana | 5 |  |  |
| Rice | 16 | Maize | 51 |  |  | Rubber | 14 |  |  |
|  |  | Banana | 50 |  |  | Oil palm | 12 |  |  |
|  |  | Rubber | 67 |  |  | Rice |  |  |  |
|  |  | Oil palm | 68 |  |  |  |  |  |  |
|  |  | Rice | 38 |  |  |  |  |  |  |

^ Data from [[1](#_ENREF_1)] for the loss of the main crops in SE Asia, except otherwise stated; * data from [[2](#_ENREF_2)] for the important weed/insect pest for each crop, except otherwise stated; ^#^ from [[3](#_ENREF_3)]; ^+^ from [[4](#_ENREF_4)].

**Table S2.** Main rodent pest species in rice production and storage in Southeast Asia [[5](#_ENREF_5),[6](#_ENREF_6)]. L = Laos, T = Thailand, M = Malaysia, I = Indonesia, P = Philippines.

| No | Species | Preharvest pest | Postharvest pest |
| --- | --- | --- | --- |
| 1 | *Bandicota indica* | L, T |  |
| 2 | *Mus caroli* | L |  |
| 3 | *Mus cervicolor* | L |  |
| 4 | *Mus musculus* |  | M, P, I |
| 5 | *Rattus argentiventer* | I, L, M, P, T |  |
| 6 | *Rattus diardii* | I, M, T, P | M, P, I |
| 7 | *Rattus exulans* | I, M, P | M, T |
| 8 | *Rattus losea* | T |  |
| 9 | *Rattus norvegicus* | I, P | M, T, P |
| 10 | *Rattus rattus* |  | T |
| 11 | *Rattus rattus diardii* |  | M, I |
| 12 | *Rattus rattus mindanensis/R.tanezumi* | P |  |

**Table S3.** Invasive species reported in Southeast Asian countries from (1) the Invasive Species Compendium [[7](#_ENREF_7)], (2) review by Peh (2010), (3) review by MacKinnon (2006), (4) the Global Invasive Species Database [[8](#_ENREF_8)]. 1 represents the presence of the species in the country as invasive.

| Species | Brunei | | | | Cambodia | | | | Indonesia | | | | Laos | | | | Malaysia | | | | Myanmar | | | | Philippines | | | | Singapore | | | | Thailand | | | | Vietnam | | | |
| --- | --- | --- | --- | --- | --- | --- | --- | --- | --- | --- | --- | --- | --- | --- | --- | --- | --- | --- | --- | --- | --- | --- | --- | --- | --- | --- | --- | --- | --- | --- | --- | --- | --- | --- | --- | --- | --- | --- | --- | --- |
|  | **(1)** | **(2)** | **(3)** | **(4)** | **(1)** | **(2)** | **(3)** | **(4)** | **(1)** | **(2)** | **(3)** | **(4)** | **(1)** | **(2)** | **(3)** | **(4)** | **(1)** | **(2)** | **(3)** | **(4)** | **(1)** | **(2)** | **(3)** | **(4)** | **(1)** | **(2)** | **(3)** | **(4)** | **(1)** | **(2)** | **(3)** | **(4)** | **(1)** | **(2)** | **(3)** | **(4)** | **(1)** | **(2)** | **(3)** | **(4)** |
| *Acacia auriculiformis* |  | 1 |  | 1 | 1 |  |  |  | 1 |  |  | 1 | 1 |  |  |  | 1 |  |  | 1 |  |  |  | 1 | 1 |  |  | 1 | 1 |  |  | 1 | 1 |  |  | 1 | 1 |  |  | 1 |
| *Acacia cincinnata* |  | 1 |  |  | 1 |  |  | 1 | 1 |  |  |  | 1 |  |  |  | 1 |  |  | 1 |  |  |  | 1 | 1 |  |  | 1 |  |  |  | 1 |  | 1 |  | 1 | 1 |  |  | 1 |
| *Acacia farnesiana* |  |  |  |  | 1 |  |  | 1 | 1 | 1 |  | 1 |  |  |  |  | 1 |  |  | 1 | 1 |  |  |  | 1 |  |  | 1 | 1 |  |  |  | 1 |  |  | 1 | 1 |  |  |  |
| *Acacia longifolia* |  |  |  |  | 1 |  |  | 1 | 1 |  |  | 1 | 1 |  |  | 1 | 1 |  |  | 1 |  |  |  |  | 1 |  |  | 1 |  |  |  | 1 |  |  |  | 1 | 1 |  |  | 1 |
| *Acacia mangium* |  |  |  |  |  | 1 |  | 1 | 1 |  |  | 1 |  | 1 |  |  | 1 |  |  | 1 | 1 |  |  |  |  |  |  |  |  |  |  |  | 1 | 1 |  | 1 |  | 1 |  | 1 |
| *Acacia nilotica* |  |  |  |  | 1 |  |  |  | 1 |  |  |  | 1 |  |  |  | 1 |  |  |  | 1 |  |  |  | 1 |  |  |  | 1 |  |  |  | 1 |  |  |  | 1 |  |  |  |
| *Acanthaster planci* |  |  |  | 1 |  |  |  | 1 |  |  |  | 1 |  |  |  | 1 |  |  |  | 1 |  |  |  | 1 |  |  |  | 1 |  |  |  | 1 |  |  |  | 1 |  |  |  | 1 |
| *Acanthospermum hispidum* |  |  |  |  |  |  |  | 1 |  |  |  | 1 |  |  |  |  |  |  |  | 1 |  |  |  |  |  |  |  | 1 |  |  |  | 1 |  |  |  | 1 | 1 |  |  | 1 |
| *Achatina fulica* |  |  |  | 1 |  |  |  | 1 |  |  |  | 1 |  |  |  |  |  |  |  | 1 |  |  |  |  |  |  |  | 1 |  |  |  | 1 |  |  |  | 1 |  |  |  | 1 |
| *Acridotheres tristis* | 1 |  |  |  | 1 |  |  |  |  |  |  |  | 1 |  |  |  | 1 |  |  |  | 1 |  |  |  | 1 |  |  |  | 1 |  |  |  | 1 |  |  |  | 1 |  |  |  |
| *Ageratina adenophora* |  |  |  |  |  |  |  |  | 1 |  |  | 1 |  |  |  |  | 1 |  |  | 1 | 1 |  |  | 1 |  |  |  |  |  |  |  |  | 1 |  |  | 1 |  |  |  |  |
| *Ageratum conyzoides* |  |  |  |  |  |  |  |  |  |  |  | 1 |  |  |  | 1 |  |  |  | 1 |  |  |  | 1 |  |  |  | 1 |  |  |  | 1 |  |  |  | 1 |  |  |  | 1 |
| *Aleurodicus dispersus* | 1 |  |  |  | 1 |  |  |  |  |  |  |  | 1 |  |  |  |  |  |  |  | 1 |  |  |  | 1 |  |  |  | 1 |  |  |  | 1 |  |  |  | 1 |  |  |  |
| *Alternanthera philoxeroides* |  |  |  |  |  |  |  |  |  |  |  |  |  |  |  |  |  |  |  | 1 |  |  |  | 1 | 1 |  |  | 1 | 1 |  |  | 1 |  |  |  | 1 |  |  |  | 1 |
| *Alternanthera sessilis* |  |  |  |  |  |  |  |  |  |  |  |  | 1 |  |  |  |  |  |  |  | 1 |  |  |  | 1 |  |  |  | 1 |  |  |  | 1 |  |  |  | 1 |  |  |  |
| *Amphibalanus improvisus* |  |  |  |  |  |  |  |  |  |  |  | 1 |  |  |  | 1 |  |  |  | 1 |  |  |  |  |  |  |  | 1 |  |  |  | 1 |  |  |  | 1 |  |  |  | 1 |
| *Annona glabra* |  |  |  |  |  |  |  |  |  |  |  | 1 |  |  |  |  | 1 |  |  | 1 |  |  |  |  |  |  |  | 1 | 1 |  |  |  |  |  |  | 1 |  |  |  |  |
| *Anoplolepis gracilipes* |  |  |  |  |  |  |  |  |  |  |  | 1 |  |  |  |  | 1 |  |  | 1 |  |  |  | 1 | 1 |  |  | 1 |  |  |  |  |  |  |  |  |  |  |  | 1 |
| *Aphis spiraecola* | 1 |  |  |  |  |  |  | 1 |  |  |  | 1 |  |  |  |  |  |  |  | 1 |  |  |  |  |  |  |  | 1 |  |  |  | 1 |  |  |  | 1 |  |  |  | 1 |
| *Austroeupatorium inulifolium* |  |  |  |  |  |  |  |  | 1 |  |  | 1 |  |  |  |  |  |  |  |  |  |  |  | 1 |  |  |  |  |  |  |  | 1 | 1 |  |  | 1 |  |  |  |  |
| *Bactrocera cucurbitae* | 1 |  |  |  |  |  |  | 1 |  |  |  | 1 |  |  |  |  |  |  |  | 1 |  |  |  |  |  |  |  | 1 |  |  |  |  |  |  |  | 1 |  |  |  | 1 |
| *Bactrocera dorsalis* |  |  |  |  |  |  |  | 1 |  |  |  | 1 |  |  |  | 1 |  |  |  | 1 |  |  |  |  |  |  |  |  |  |  |  |  |  |  |  | 1 |  |  |  | 1 |
| *Banana bunchy top virus* |  |  |  |  |  |  |  |  |  |  |  |  |  |  |  |  |  | 1 |  | 1 |  |  |  |  |  |  |  |  | 1 |  |  | 1 | 1 |  |  |  |  |  |  |  |
| *Beak and feather disease virus* |  |  |  |  | 1 |  |  |  | 1 |  |  |  |  |  |  |  |  |  |  |  | 1 |  |  |  | 1 |  |  |  | 1 |  |  |  |  |  |  |  | 1 |  |  |  |
| *Bemisia tabaci* |  |  |  |  |  |  |  | 1 |  |  |  | 1 |  |  |  |  |  |  |  | 1 |  |  |  |  |  |  |  | 1 |  |  |  |  |  |  |  | 1 |  |  |  | 1 |
| *Bidens pilosa* |  |  |  |  |  |  |  |  | 1 |  |  | 1 |  |  |  |  |  |  |  |  | 1 |  |  |  |  |  |  | 1 |  |  |  | 1 |  |  |  |  |  |  |  | 1 |
| *Bougainvillea spectabilis* |  |  |  |  |  |  |  |  |  |  |  | 1 |  |  |  |  |  |  |  | 1 |  |  |  |  |  |  |  |  |  |  |  | 1 |  |  |  | 1 |  |  |  |  |
| *Brontispa longissima* |  |  |  |  | 1 |  |  |  |  |  |  |  | 1 |  |  |  |  |  |  |  | 1 |  |  |  |  |  |  |  |  |  |  |  | 1 |  |  |  | 1 |  |  |  |
| *Bufo marinus* |  |  |  |  |  |  |  |  |  |  |  |  |  |  |  |  |  |  |  | 1 |  |  |  |  |  |  |  |  | 1 | 1 |  | 1 |  |  |  |  |  |  |  |  |
| *Bugula neritina* |  |  |  |  |  |  |  |  |  |  |  |  |  |  |  |  |  |  |  |  | 1 |  |  |  | 1 |  |  |  |  |  |  |  | 1 |  |  |  | 1 |  |  |  |
| *Cabomba caroliniana* |  |  |  |  |  |  |  |  |  |  |  |  |  |  |  |  |  |  |  |  |  |  |  |  |  |  |  |  | 1 |  |  | 1 | 1 |  |  |  |  |  |  | 1 |
| *Cacatua sulphurea* |  |  |  |  |  |  |  |  |  |  |  | 1 |  |  |  |  |  |  |  | 1 |  |  |  |  |  |  |  | 1 |  |  |  |  |  |  |  | 1 |  |  |  | 1 |
| *Caesalpinia pulcherrima* |  |  |  |  |  |  |  |  |  |  |  | 1 |  |  |  |  |  |  |  | 1 |  |  |  |  |  |  |  | 1 |  |  |  | 1 |  |  |  | 1 |  |  |  |  |
| *Candidatus Liberibacter asiaticus* |  |  |  |  |  |  |  | 1 |  |  |  | 1 |  |  |  |  |  |  |  | 1 |  |  |  |  |  |  |  | 1 |  |  |  | 1 |  |  |  |  |  |  |  |  |
| *Cecropia peltata* |  |  |  |  |  |  |  |  | 1 |  |  |  |  |  |  |  | 1 |  |  |  | 1 |  |  |  | 1 |  |  |  |  |  |  |  | 1 |  |  |  |  |  |  |  |
| *Cenchrus echinatus* |  |  |  |  |  |  |  |  |  |  |  |  |  |  |  |  |  |  |  |  |  |  |  |  |  |  |  |  | 1 | 1 |  | 1 |  |  |  |  |  |  |  |  |
| *Cenchrus polystachios* |  |  |  |  |  |  |  |  |  |  |  | 1 |  |  |  |  |  |  |  |  |  |  |  | 1 |  |  |  |  |  |  |  | 1 |  |  |  | 1 |  |  |  |  |
| *Chromolaena odorata* | 1 |  |  |  |  |  |  |  | 1 |  |  |  |  |  |  |  | 1 |  |  |  |  |  |  |  | 1 |  |  |  |  |  |  |  |  |  |  |  | 1 |  |  |  |
| *Cichlasoma urophthalmus* |  |  |  |  |  |  |  | 1 |  |  |  |  |  |  |  |  |  |  |  |  |  |  |  |  |  |  |  | 1 |  |  |  |  |  |  |  | 1 |  |  |  | 1 |
| *Clarias batrachus* |  |  |  |  |  |  |  |  |  |  |  |  |  |  |  |  | 1 |  |  |  |  |  |  |  |  |  |  |  | 1 |  |  |  | 1 |  |  |  | 1 |  |  |  |
| *Claviceps africana* |  |  |  |  |  |  |  |  |  |  |  | 1 |  |  |  |  |  |  |  | 1 |  |  |  | 1 |  |  |  | 1 |  |  |  |  |  |  |  |  |  |  |  |  |
| *Clidemia hirta* | 1 |  |  |  |  |  |  |  |  | 1 |  | 1 |  |  |  |  |  |  |  |  |  |  |  |  |  |  |  |  |  | 1 |  |  |  |  |  |  |  |  |  |  |
| *Coccinea indica* |  |  |  |  |  |  |  |  |  |  |  |  |  |  |  |  |  |  |  |  |  |  |  |  |  |  |  |  | 1 |  |  |  |  |  |  |  |  |  |  |  |
| *Coconut cadang-cadang viroid* |  |  |  |  |  |  |  |  | 1 |  |  |  |  |  |  |  |  |  |  |  | 1 |  |  |  |  |  |  |  |  |  |  |  | 1 |  |  |  |  |  |  |  |
| *Columba livia* |  |  |  |  |  |  |  |  | 1 |  |  |  |  |  |  |  |  |  |  |  |  |  |  |  | 1 |  |  |  |  |  |  |  | 1 |  |  |  |  |  |  |  |
| *Common myna* |  |  |  |  |  |  |  |  | 1 |  |  | 1 |  |  |  |  |  |  |  |  |  |  |  |  |  |  |  |  |  |  |  |  |  |  |  |  |  |  |  |  |
| *Cordylophora* |  |  |  |  |  |  |  |  | 1 |  |  |  |  |  |  |  | 1 |  |  |  |  |  |  |  |  |  |  |  |  |  |  |  |  |  |  |  |  |  |  |  |
| *Corvus splendens* |  |  |  |  |  |  |  |  |  |  |  | 1 |  |  |  |  |  |  |  |  |  |  |  |  |  |  |  | 1 |  |  |  |  |  |  |  | 1 |  |  |  |  |
| *Cynodon dactylon* |  |  |  |  |  |  |  |  |  |  |  | 1 |  |  |  |  |  |  |  |  |  |  |  |  |  |  |  | 1 |  |  |  |  |  |  |  |  |  |  |  |  |
| *Cyperus rotundus* |  |  |  |  |  |  |  |  | 1 |  |  |  |  |  |  |  | 1 |  |  |  |  |  |  |  | 1 |  |  |  |  |  |  |  |  |  |  |  |  |  |  |  |
| *Cyprinus carpio* |  |  |  |  |  |  |  |  |  |  |  |  |  |  |  |  |  |  |  |  |  |  |  |  | 1 |  |  |  |  |  |  |  | 1 |  |  |  | 1 |  |  |  |
| *Desmostachya bipinnata* |  |  |  |  |  |  |  |  |  |  |  |  |  |  |  |  |  |  |  |  |  |  |  |  | 1 |  |  |  | 1 |  |  |  |  |  |  |  | 1 |  |  |  |
| *Diaphorina citri* |  |  |  |  |  |  |  |  | 1 |  |  |  |  |  |  |  |  |  |  |  |  |  |  |  |  |  |  |  |  |  |  |  | 1 |  |  |  | 1 |  |  |  |
| *Eichhomia crassipes* |  |  |  |  |  |  |  |  |  |  |  | 1 |  |  |  |  |  |  |  |  |  |  |  |  |  |  |  | 1 |  |  |  |  |  |  |  |  |  |  |  |  |
| *Eucalyptus* |  |  |  |  |  |  |  |  |  |  |  | 1 |  |  |  |  |  |  |  |  |  |  |  |  |  |  |  | 1 |  |  |  |  |  |  |  | 1 |  |  |  |  |
| *Eugenia uniflora* |  |  |  |  |  |  |  | 1 |  |  |  |  |  |  |  |  |  |  |  | 1 |  |  |  |  |  |  |  | 1 |  |  |  |  |  |  |  |  |  |  |  |  |
| *Euglandina rosea* |  |  |  |  |  |  |  |  |  |  |  |  |  |  |  |  |  |  |  |  |  |  |  |  |  |  |  |  |  |  |  |  |  |  |  |  | 1 |  |  | 1 |
| *Euplatypus parallelus* |  |  |  |  |  |  |  |  |  |  |  | 1 |  |  |  |  |  |  |  |  |  |  |  |  |  |  |  | 1 |  |  |  |  |  |  |  |  |  |  |  |  |
| *Eurasian tree sparrow* |  |  |  |  |  |  |  |  |  |  |  | 1 |  |  |  |  |  |  |  |  |  |  |  |  |  |  |  |  |  |  |  |  |  |  |  | 1 |  |  |  |  |
| *Fusarium oxysporum f.sp. cubense* | 1 |  |  |  |  |  |  |  |  |  |  |  |  |  |  |  |  |  |  |  |  |  |  |  | 1 |  |  | 1 |  |  |  |  |  |  |  |  |  |  |  |  |
| *Gambusia holbrooki* |  |  |  |  |  |  |  |  |  |  |  |  |  |  |  |  |  |  |  |  |  |  |  |  |  |  |  |  |  | 1 |  |  |  |  |  |  |  |  |  |  |
| *Hypophthalmichthys molitrix* |  |  |  |  | 1 |  |  |  |  |  |  |  |  |  |  |  |  |  |  |  | 1 |  |  |  |  |  |  |  |  |  |  |  |  |  |  |  |  |  |  |  |
| *Hypophthalmichthys nobilis* |  |  |  |  |  |  |  |  | 1 |  |  | 1 |  |  |  |  |  |  |  |  |  |  |  |  |  |  |  |  |  |  |  |  |  |  |  |  |  |  |  |  |
| *Icerya purchasi* |  |  |  |  |  |  |  |  |  |  |  |  |  |  |  |  |  |  |  |  |  |  |  |  |  | 1 |  |  |  |  |  |  |  |  |  |  |  |  |  |  |
| *Imperata cylindrica* |  |  |  |  |  |  |  |  |  |  |  | 1 |  |  |  |  |  |  |  |  |  |  |  |  |  |  |  |  |  |  |  |  |  |  |  |  |  |  |  |  |
| *Ipomoea cairica* |  |  |  |  |  |  |  | 1 |  |  |  |  |  |  |  |  |  |  |  |  |  |  |  |  |  |  |  | 1 |  |  |  |  |  |  |  |  |  |  |  |  |
| *Ipomoea carnea* |  |  |  |  |  |  |  |  |  |  |  |  |  |  |  |  |  |  |  |  |  |  |  |  |  |  |  |  |  |  |  |  | 1 |  |  | 1 |  |  |  |  |
| *Ipomoea aquatica* |  |  |  |  |  |  |  |  |  |  |  |  |  |  |  |  |  |  |  |  |  |  |  |  |  |  |  | 1 |  |  |  |  |  |  |  | 1 |  |  |  |  |
| *Jatropha gossypiifolia* |  |  |  |  |  |  |  |  |  |  |  |  |  |  |  |  |  |  |  |  |  |  |  |  |  | 1 |  |  |  |  |  |  |  |  |  |  |  |  |  |  |
| *Lantana camara* | 1 |  |  |  |  |  |  | 1 |  |  |  |  |  |  |  |  |  |  |  |  |  |  |  |  |  |  |  |  |  |  |  |  |  |  |  |  |  |  |  | 1 |
| *Leafminers* |  |  |  |  |  |  |  |  |  |  |  |  |  |  |  |  | 1 |  |  |  |  |  |  |  |  |  |  |  |  |  |  |  | 1 |  |  |  |  |  |  |  |
| *Lepidium virginicum* |  |  |  |  |  |  |  |  |  |  |  |  |  |  |  |  |  |  |  |  |  |  |  |  |  |  |  |  |  |  |  |  |  |  |  |  |  |  |  |  |
| *Leucaena leucocephala* |  |  |  |  |  |  |  |  |  |  |  |  |  |  |  |  |  |  |  |  |  |  |  |  | 1 |  |  | 1 |  |  |  |  |  |  |  |  |  |  |  |  |
| *Limnocharis flava* |  |  |  |  |  |  |  |  |  |  |  |  |  |  |  |  |  |  |  |  |  |  |  |  |  |  |  |  | 1 |  |  | 1 |  |  |  |  |  |  |  |  |
| *Lissachatina fulica* |  |  |  |  |  |  |  |  |  |  |  |  |  |  |  |  |  |  |  |  |  |  |  |  |  |  |  |  | 1 |  |  |  |  |  |  |  |  |  |  |  |
| *Ludwigia peruviana* |  |  |  |  |  |  |  | 1 |  |  |  | 1 |  |  |  |  |  |  |  |  |  |  |  |  |  |  |  |  |  |  |  |  |  |  |  |  |  |  |  |  |
| *Mikania micrantha* |  |  |  |  |  |  |  |  |  |  |  | 1 |  |  |  |  |  |  |  |  |  |  |  |  |  |  |  |  |  |  |  |  |  |  |  | 1 |  |  |  |  |
| *Mimosa diplotricha* |  |  |  |  |  |  |  |  |  |  |  |  |  |  |  |  |  |  |  | 1 |  |  |  |  |  |  |  |  |  |  |  | 1 |  |  |  |  |  |  |  |  |
| *Mimosa pigra* |  |  |  |  |  |  |  |  |  |  |  |  |  |  |  |  |  |  |  |  |  |  |  |  |  |  |  |  |  |  |  |  | 1 |  |  | 1 |  |  |  |  |
| *Mimosa pudica* |  |  |  |  |  |  |  |  |  |  |  |  |  |  |  |  |  |  |  |  |  |  |  |  |  |  |  |  |  |  |  |  |  |  |  |  |  |  |  |  |
| *Murdannia nudiflora* |  |  |  |  |  |  |  |  | 1 |  |  |  |  |  |  |  |  |  |  |  |  |  |  |  |  |  |  |  |  |  |  |  |  |  |  |  |  |  |  |  |
| *Mycosphaerella fijiensis* |  |  |  |  |  |  |  |  |  |  |  |  |  |  |  |  |  |  |  |  |  |  |  | 1 |  |  |  |  |  |  |  |  |  |  |  |  |  |  |  |  |
| *Myocastor coypus* |  |  |  |  |  |  |  |  |  |  |  |  |  |  |  |  |  |  |  |  |  |  |  |  |  |  |  |  |  |  |  |  |  |  |  |  |  |  |  |  |
| *Myriophyllum aquaticum* |  |  |  |  |  |  |  |  | 1 |  |  |  |  |  |  |  |  |  |  |  |  |  |  |  |  |  |  |  |  |  |  |  |  |  |  |  |  |  |  |  |
| *Nipaecoccus nipae* |  |  |  |  |  |  |  |  | 1 |  |  |  |  |  |  |  |  |  |  |  |  |  |  |  |  |  |  |  |  |  |  |  |  |  |  |  |  |  |  |  |
| *Opuntia monacantha* |  |  |  |  |  |  |  |  |  |  |  |  |  |  |  | 1 |  |  |  |  |  |  |  |  |  |  |  |  |  |  |  |  |  |  |  |  |  |  |  |  |
| *Oreochromis niloticus* |  |  |  |  |  |  |  |  |  |  |  |  |  |  |  |  |  |  |  |  |  |  |  |  |  |  |  |  |  |  |  |  |  |  |  |  | 1 |  |  |  |
| *Oreochromis spp.* |  |  |  |  |  |  |  |  |  |  |  |  |  |  |  |  |  |  |  |  |  |  |  |  |  |  |  |  |  |  |  |  |  |  |  |  |  |  |  |  |
| *Oxalis corniculata* |  |  |  |  |  |  |  |  |  |  |  |  |  |  |  |  |  |  |  |  |  |  |  |  |  |  |  |  |  |  |  |  |  |  |  |  |  |  |  |  |
| *Oxalis latifolia* |  |  |  |  |  |  |  |  |  |  |  |  |  |  |  |  |  |  |  | 1 |  |  |  |  |  |  |  |  |  |  |  |  |  |  |  |  |  |  |  |  |
| *Pachystachys coccinea* |  |  |  |  |  |  |  |  |  |  |  |  |  |  |  |  |  |  |  |  |  |  |  |  |  |  |  |  |  |  |  |  |  |  |  |  |  |  |  |  |
| *Padda oryzivora* |  |  |  |  |  |  |  |  |  |  |  |  |  |  |  |  |  |  |  |  |  |  |  |  |  |  |  |  |  |  |  |  |  |  |  |  |  |  |  |  |
| *Panicum repens* |  |  |  |  |  |  |  |  |  |  |  | 1 |  |  |  |  |  |  |  |  |  |  |  |  |  |  |  |  |  |  |  |  |  |  |  |  |  |  |  |  |
| *Paradoxurus hermaphroditus* |  |  |  |  |  |  |  |  |  |  |  |  |  |  |  |  |  |  |  | 1 |  |  |  |  |  |  |  |  |  |  |  |  |  |  |  |  |  |  |  |  |
| *Paratrechina longicornis* | 1 |  |  |  |  |  |  |  |  |  |  |  |  |  |  |  |  |  |  |  |  |  |  |  |  |  |  |  |  |  |  |  |  |  |  | 1 |  |  |  |  |
| *Parthenium hysterophorus* |  |  |  |  |  |  |  |  |  |  |  |  |  |  |  |  |  |  |  |  |  |  |  |  |  |  |  |  |  |  |  | 1 |  |  |  |  |  |  |  |  |
| *Paspalum vaginatum* |  |  |  |  |  |  |  |  |  |  |  |  |  |  |  |  |  |  |  |  |  |  |  |  |  |  |  |  |  |  |  |  | 1 |  |  |  |  |  |  |  |
| *Passiflora edulis* |  |  |  |  |  |  |  |  |  |  |  |  |  |  |  |  |  |  |  |  |  |  |  |  |  |  |  |  |  |  |  |  |  |  |  |  |  |  |  |  |
| *Passiflora foetida* |  |  |  |  |  |  |  |  |  |  |  |  |  |  |  |  |  |  |  |  |  |  |  |  | 1 |  |  |  |  |  |  |  |  |  |  |  |  |  |  |  |
| *Paulownia tomentosa* |  |  |  |  |  |  |  |  |  |  |  |  |  |  |  |  |  |  |  |  |  |  |  |  |  |  |  |  |  | 1 |  |  |  |  |  |  |  |  |  |  |
| *Pennisetum pedicellatum* |  |  |  |  |  |  |  |  |  |  |  |  |  |  |  |  |  |  |  |  |  |  |  |  | 1 |  |  |  |  |  |  |  |  |  |  |  |  |  |  |  |
| *Periplaneta americana* |  |  |  |  |  |  |  |  |  |  |  |  |  |  |  |  |  |  |  |  |  |  |  |  |  |  |  |  |  |  |  |  |  |  |  |  |  |  |  |  |
| *Pheidole megacephala* |  |  |  |  |  |  |  |  |  |  |  | 1 |  |  |  |  |  |  |  |  |  |  |  |  |  |  |  |  |  |  |  |  |  |  |  |  |  |  |  |  |
| *Phenacoccus solenopsis* |  |  |  |  |  |  |  |  |  |  |  |  |  |  |  |  |  |  |  |  |  |  |  |  |  |  |  |  |  |  |  |  |  |  |  |  |  |  |  |  |
| *Physalis peruviana* |  |  |  |  |  |  |  |  |  |  |  | 1 |  |  |  |  |  |  |  |  |  |  |  |  |  |  |  |  |  |  |  |  |  |  |  |  |  |  |  |  |
| *Piper aduncum* |  |  |  |  |  |  |  |  |  |  |  |  |  |  |  |  |  | 1 |  |  |  |  |  |  |  |  |  |  |  |  |  |  |  |  |  |  |  |  |  |  |
| *Pistia stratiotes* |  |  |  |  |  |  |  |  |  |  |  |  |  |  |  |  |  |  |  |  |  |  |  |  |  |  |  | 1 |  |  |  |  |  |  |  |  |  |  |  |  |
| *Platydemus manokwari* |  |  |  |  |  |  |  |  |  |  |  |  |  |  |  |  |  |  |  |  |  |  |  |  |  |  |  |  |  |  |  |  |  |  |  |  |  |  |  |  |
| *Poecilia reticulata* |  |  |  |  |  |  |  |  |  |  |  |  |  |  |  |  |  |  |  |  |  |  |  |  |  |  |  |  |  |  |  |  |  |  |  |  |  |  |  |  |
| *Polygonum persicaria* |  |  |  |  |  |  |  |  |  |  |  |  |  |  |  |  |  |  |  |  |  |  |  |  | 1 |  |  |  |  |  |  |  |  |  |  |  |  |  |  |  |
| *Pomacea canaliculata* |  |  |  |  |  |  |  |  |  |  |  |  |  |  |  |  |  |  |  |  |  |  |  |  |  |  |  |  |  |  |  |  |  |  |  |  |  | 1 |  |  |
| *Pomacea insularum* |  |  |  |  |  |  |  |  |  |  |  |  |  |  |  |  |  |  |  |  |  |  |  |  |  |  |  | 1 |  |  |  |  |  |  |  |  |  |  |  |  |
| *Pseudorasbora parva* |  |  |  |  |  |  |  |  |  |  |  | 1 |  |  |  |  |  |  |  |  |  |  |  |  |  |  |  |  |  |  |  |  |  |  |  |  |  |  |  |  |
| *Psidium guajava* |  |  |  |  |  |  |  |  |  |  |  | 1 |  |  |  |  |  |  |  |  |  |  |  |  |  |  |  |  |  |  |  |  |  |  |  |  |  |  |  |  |
| *Psittacula krameri* |  |  |  |  |  |  |  |  |  |  |  |  |  |  |  |  |  |  |  |  |  |  |  |  | 1 |  |  |  |  |  |  |  |  |  |  |  |  |  |  |  |
| *Pterygoplichthys disjunctivus* |  |  |  |  |  |  |  |  |  |  |  |  |  |  |  |  |  |  |  |  |  |  |  |  |  |  |  |  |  |  |  |  |  |  |  |  |  |  |  |  |
| *Pterygoplichthys pardalis* |  |  |  |  |  |  |  |  |  |  |  |  |  |  |  |  |  |  |  |  |  |  |  |  |  |  |  | 1 |  |  |  |  |  |  |  |  |  |  |  |  |
| *Pterygoplichthys spp.* |  |  |  |  |  |  |  |  |  |  |  | 1 |  |  |  |  |  |  |  |  |  |  |  |  |  |  |  |  |  |  |  |  |  |  |  |  |  |  |  |  |
| *Quadrastichus erythrinae* |  |  |  |  |  |  |  |  |  |  |  |  |  |  |  |  |  |  |  |  |  |  |  |  |  |  | 1 |  |  |  |  |  |  |  |  |  |  |  |  |  |
| *Rana catesbeiana* |  |  |  |  |  |  |  |  |  |  |  |  |  |  |  |  |  |  |  |  |  |  |  |  |  |  |  |  |  |  |  |  |  |  |  |  |  |  |  |  |
| *Rattus exulans* |  |  |  |  |  |  |  |  |  |  |  |  |  |  |  |  |  |  |  |  |  |  |  |  |  |  |  |  |  |  |  |  |  |  |  |  |  |  |  |  |
| *Rattus norvegicus* |  |  |  |  |  |  |  |  |  |  |  |  |  |  |  |  |  |  |  |  |  |  |  |  |  |  |  |  |  |  |  |  |  |  |  |  | 1 |  |  |  |
| *Rattus rattus* |  |  |  |  |  |  |  |  |  |  |  |  |  |  |  |  |  |  |  | 1 |  |  |  |  |  |  |  |  |  |  |  |  |  |  |  |  |  |  |  |  |
| *Red avadavat* |  |  |  |  |  |  |  |  |  | 1 |  |  |  |  |  |  |  |  |  |  |  |  |  |  |  |  |  |  |  |  |  |  |  |  |  |  |  |  |  |  |
| *Ricinus communis* |  |  |  |  |  |  |  |  |  |  |  |  |  |  |  |  |  |  |  |  |  |  |  |  |  |  |  |  |  |  |  |  |  |  |  |  |  |  |  | 1 |
| *Salmo salar* |  |  |  |  |  |  |  |  |  |  |  |  |  |  |  |  |  |  |  |  |  |  |  |  |  |  |  |  |  |  |  |  |  |  |  |  |  |  |  |  |
| *Salvinia molesta* |  |  |  |  |  |  |  |  |  |  |  | 1 |  |  |  |  |  |  |  |  |  |  |  |  |  |  |  |  |  |  |  |  |  |  |  |  |  |  |  |  |
| *Scaly-breasted munia* |  |  |  |  |  |  |  |  |  |  |  |  |  |  |  |  |  |  |  |  |  |  |  |  |  |  |  |  |  |  |  |  |  |  |  |  |  |  |  |  |
| *Senna obtusifolia* |  |  |  |  |  |  |  |  |  |  |  |  |  |  |  |  |  |  |  |  |  |  |  |  | 1 |  |  |  |  |  |  |  |  |  |  |  |  |  |  |  |
| *Setaria verticillata* |  |  |  |  |  |  |  |  |  |  |  |  | 1 |  |  |  |  |  |  |  |  |  |  |  |  |  |  |  |  |  |  |  |  |  |  |  |  |  |  |  |
| *Solenopsis geminata* |  |  |  |  |  |  |  |  |  |  |  |  |  |  |  |  |  |  |  | 1 |  |  |  |  |  |  |  |  |  |  |  |  |  |  |  |  |  |  |  |  |
| *Solenopsis invicta* |  |  |  |  |  |  |  |  |  |  |  |  |  |  |  |  |  |  |  |  |  |  |  |  |  |  |  |  |  |  |  |  | 1 |  |  |  |  |  |  |  |
| *Sorghum halepense* |  |  |  |  |  |  |  |  |  |  |  |  |  |  |  |  |  |  |  |  |  |  |  |  |  |  |  | 1 |  |  |  |  |  |  |  |  |  |  |  |  |
| *Spathodea campanulata* |  |  |  |  |  |  |  |  |  |  |  |  |  |  |  |  |  |  |  |  |  |  |  |  |  |  |  | 1 |  |  |  |  |  |  |  |  |  |  |  |  |
| *Spodoptera litura* | 1 |  |  |  |  |  |  |  |  |  |  |  |  |  |  |  |  |  |  |  |  |  |  |  |  |  |  | 1 |  |  |  |  |  |  |  |  |  |  |  |  |
| *Striga asiatica* |  |  |  |  |  |  |  |  |  |  |  |  |  |  |  |  |  |  |  |  |  |  |  |  |  |  |  |  |  |  |  |  |  |  |  |  |  |  |  |  |
| *Syngonium podophyllum* |  |  |  |  |  |  |  |  |  |  |  |  |  |  |  |  |  |  |  |  |  |  |  |  |  | 1 |  |  |  |  |  |  |  |  |  |  |  |  |  |  |
| *Tapinoma melanocephalum* |  |  |  |  |  |  |  |  |  |  |  |  |  |  |  |  |  |  |  |  |  |  |  |  |  |  |  |  |  |  |  | 1 |  |  |  |  |  |  |  |  |
| *Technomyrmex albipes* |  |  |  |  |  |  |  |  |  |  |  |  |  |  |  |  |  |  |  |  |  |  |  |  |  | 1 |  |  |  |  |  |  |  |  |  |  |  |  |  |  |
| *Tecoma stans* |  |  |  |  |  |  |  |  |  |  |  |  |  |  |  |  |  |  |  |  |  |  |  |  | 1 |  |  |  |  |  |  |  |  |  |  |  |  |  |  |  |
| *Thevetia peruviana* |  |  |  |  |  |  |  |  |  |  |  |  |  |  |  |  |  |  |  |  |  |  |  |  |  |  |  |  | 1 |  |  |  |  |  |  |  |  |  |  |  |
| *Thunbergia grandiflora* |  |  |  |  |  |  |  |  |  |  |  |  |  |  |  |  |  |  |  |  |  |  |  |  |  |  |  |  |  |  |  | 1 |  |  |  |  |  |  |  |  |
| *Tilapia mossambica* |  |  |  |  |  |  |  |  |  |  |  |  |  |  |  |  |  |  |  | 1 |  |  |  |  |  |  |  |  |  |  |  |  |  |  |  |  |  |  |  |  |
| *Trachemys scripta elegans* |  |  |  |  |  |  |  |  |  |  |  |  |  |  |  |  |  |  |  |  |  |  |  |  | 1 |  |  |  |  |  |  |  |  |  |  |  |  |  |  |  |
| *Trichoglossus haematodus* |  |  |  |  |  |  |  |  |  |  |  | 1 |  |  |  |  |  |  |  |  |  |  |  |  |  |  |  |  |  |  |  |  |  |  |  |  |  |  |  |  |
| *Urochloa mutica* |  |  |  |  |  |  |  |  |  |  |  |  |  |  |  |  |  |  |  |  |  |  |  |  |  |  |  |  |  |  |  |  |  |  |  |  |  |  |  |  |
| *Verbena brasiliensis* |  |  |  |  |  |  |  |  |  |  |  |  |  |  |  |  |  |  |  |  |  |  |  |  |  |  |  |  |  |  |  |  |  |  |  |  |  |  |  |  |
| *Vibrio cholerae* |  |  |  |  |  |  |  |  |  |  |  |  |  |  |  |  |  |  |  |  |  |  |  |  |  |  |  |  |  |  |  |  |  |  |  |  |  |  |  |  |
| *White-vented myna* |  |  |  |  |  |  |  |  |  |  |  | 1 |  |  |  |  |  |  |  |  |  |  |  |  |  |  |  |  |  |  |  |  |  |  |  |  |  |  |  |  |
| *Xyleborinus saxesenii* |  |  |  |  |  |  |  |  |  |  |  |  |  |  |  |  |  |  |  |  |  |  |  |  |  |  |  |  |  | 1 |  |  |  |  |  |  |  |  |  |  |
| *Xyleborus volvulus* |  |  |  |  |  |  |  |  |  |  |  |  |  |  |  |  |  |  |  |  |  |  |  |  | 1 |  |  |  |  |  |  |  |  |  |  |  |  |  |  |  |

**Table S4.** List of projects related to NIS in Southeast Asia

| No | Project title | Time | Project budget (US$) |
| --- | --- | --- | --- |
| 1 | Control of Newcastle disease and identification of major constraints in village chicken production systems in Myanmar | 1/2003 – 5/2008 | 553,356 |
| 2 | Identification of policy responses to minimise negative socio-economic impacts of an avian influenza epidemic in Indonesia | 4/2006 – 6/2008 | 399,641 |
| 3 | The epidemiology, pathogenesis and control of highly pathogenic avian influenza (HPAI) in ducks in Indonesia and Vietnam | 3/2006 – 12/2010 | 1,501,343 |
| 4 | Vaccination of Malaysian village poultry with an avirulent Australian Newcastle disease virus | 1/1984 – 6/1987 | 507,000 |
| 5 | Control of Newcastle disease in village chickens with oral V4 vaccine | 1/1988 -12/1991 | 1,101,564 |
| 6 | Biological control of *Chromolaena odorata* in Indonesia and Papua New Guinea | 7/1997 – 3/2007 | 1,055,012 |
| 7 | *Liriomyza huidobrensis* leaf miner: developing effective pest management strategies for Indonesia and Australia | 1/2001 – 12/2004 | 614,261 |
| 8 | Control of giant sensitive plant *Mimosa pigra* | 7/1983 – 6/1986 | 594,588 |
| 9 | Control of giant sensitive plant (*Mimosa pigra*) in Thailand | 7/1987 – 6/1990 | 547,353 |
| 10 | Biological control of water hyacinth in Thailand | 7/1990 – 12/1994 | 241,389 |
| 11 | Biological control of *Chromolaena odorata* in Indonesia and the Philippines | 1/1993 – 6/1997 | 536,456 |
| 12 | Biological control of giant sensitive plant (*Mimosa pigra*) in Southeast Asia | 1/1995 – 12/1998 | 291,126 |
| 13 | Biological control of water hyacinth in Southeast Asia | 1/1995 – 12/1999 | 816,030 |
| 14 | Tonle Sap Conservation Project | 7/2004 – 12/2011 | 1,091,300 |
| 15 | Mekong River Basin Wetland Biodiversity Conservation and Sustainable Use Program | 7/2004 – 11/2009 | 170,000 |
| 16 | Removing Barriers to Invasive Species Management in Production and Protection Forests in SE Asia | 12/2011 – 11/2015 | 7,080,221 |
| 17 | TCP/RAS/3311: Capacity Building for Spread Prevention and Management of Cassava Pink Mealybug in the Greater Mekong Subregion | 1/2011 – 1/2012 | 491,000 |
| 18 | OSRO/INS/103/USA: Enhancing the capacity of the Government of Indonesia and partners to control Highly Pathogenic Avian Influenza (HPAI) | 1/2011 – 1/2012 | 8,200,000 |
| 19 | OSRO/INS/604/USA: Expansion of the Avian Influenza Participatory Disease Surveillance and Response Program in Indonesia | 1/2006 – 1/2012 | 44,200,000 |
| 20 | OSRO/INT/805/USA: Developing and Maintaining Public-Private Partnerships for the Prevention and Control of Highly Pathogenic Avian Influenza H5N1 | 1/2009 – 1/2012 | 2,457,206 |
| 21 | OSRO/MYA/702/USA: Immediate Technical Assistance to Strengthen Emergency Preparedness for Highly Pathogenic Avian Influenza (HPAI) (Grant N. GHA-G-00-06-00001) | 1/2008 – 1/2012 | 2,054,000 |
| 22 | FAO/TCP/Integrated management approach for the golden snail in rice paddles in Vietnam | 1/1998 – 1/2000 | 250,000 |
| 23 | FAO/TCP/Technical support in management of *Brontispa longissima* in Vietnam | 1/2001 – 1/2003 | 500,000 |

**References**

1. Oerke EC, Dehne HW, Schönbeck F, Weber A (1994) Crop production and crop protection: estimated losses in major food and cash crops. Elsevier.

2. Waterhouse DF (1993) The major anthropod pests and weeds of agriculture in Southeast Asia: Distribution, importance and origin. Canberra, Australia: Australian Centre for International Agriculture Research.

3. Melifonwu A (1994) Weeds and their control in cassava. African Crop Science Journal 2: 519 - 530.

4. Pathak M, Khan Z (1994) Insect pests of rice.

5. Singleton GR, Petch DA (1994) A review of the biology and management of rodent pests in Southeast Asia. Canberra, Australia. 65 p.

6. Singleton GR (2003) Impacts of rodents on rice production in Asia. IRRI Discuss Pap Ser 45: 30.

7. CABI (2012) Invasive Species Compendium. Centre for Agriculture and Biosciences International.

8. The Invasive Species Specialist Group (2012) Global Invasive Species Database
